# Study and Simulation Analysis of Microwave Heating Performance of Magnetite Concrete Based on Random Aggregate Modeling

**DOI:** 10.3390/ma18061333

**Published:** 2025-03-18

**Authors:** Guoyu Li, Zhenfu Chen, Qiongfang Wu, Dan Wu, Qiuwang Tao, Pinyu Zou, Yizhi Liu

**Affiliations:** 1 School of Civil Engineering, University of South China, Hengyang 421001, China; 20222009110559@stu.usc.edu.cn (G.L.); chenzhenfu@usc.edu.cn (Z.C.); 2017000067@usc.edu.cn (D.W.); taoqiuwang@usc.edu.cn (Q.T.); nhzpy1987@sina.com (P.Z.); 2018000039@usc.edu.cn (Y.L.); 2 China Nuclear Industry Key Laboratory of High-Performance Concrete, University of South China, Hengyang 421001, China; 3 Hunan Provincial Key Laboratory of High-Performance Special Concrete, University of South China, Hengyang 421001, China

**Keywords:** random aggregate model, magnetite concrete, microwave heating, thermal evolution

## Abstract

Radiation-shielding concrete, widely used in protective structures because of its effective shielding properties, employs magnetite aggregates to achieve higher compressive strength than conventional concrete. However, prolonged exposure to high temperatures leads to mechanical degradation. This study investigates the thermal evolution of magnetite concrete under microwave heating across varying temperatures (38–800 °C). A microwave oven was utilized for heating, and COMSOL Multiphysics was employed to establish an electromagnetic-thermal-mechanical coupled model, analyzing surface characteristics, temperature distribution, stress-strain behavior, and residual compressive strength. Results indicate that internal temperatures exceed surface temperatures during microwave heating, with a maximum temperature difference surpassing 150 °C at 800 °C. Compressive stresses predominantly arise in the mortar, while tensile stresses concentrate in aggregates and the interface transition zone, causing stress concentration. Mortar exhibits greater deformation than aggregates as temperatures increase. Simulated and experimental residual compressive strengths show strong agreement, with a maximum deviation of 7.58%. The most rapid mechanical deterioration occurs at 450–600 °C, marked by a residual compressive strength decline of 0.07 MPa/°C and the formation of penetrating cracks.

## 1. Introduction

Radiation-resistant concrete has become a commonly used building protection material in areas such as medical facilities and human defense projects due to its high aggregate density, radiation resistance, and ability to reduce the cross-sectional dimensions of components significantly [1]. Researchers have incorporated activators such as fly ash and nano-silica into concrete to enhance its structural toughness and strength [2,3]. Currently radioprotective concrete is prepared using high-density materials acting as aggregates, mixed with an admixture containing a certain amount of water of crystallization and hydride additives. This ensures that the concrete has good durability and mechanical properties but also effectively attenuates radiation such as x-rays, γ-rays, and neutrons and has a good shielding effect [4,5,6]. Studies have shown that concrete prepared with mineral sands and gravels containing large amounts of crystalline water, such as limonite, barite, and magnetite, as aggregates not only has a large apparent density, which improves the compressive strength of the concrete but also possesses sufficient H-elements, which provide good radiation shielding against gamma rays and neutron flow [7,8,9,10].

However, radiation-proof concrete serves in high temperature and radiation environments all year round; mortar and aggregate produce an expansion effect after being warmed, resulting in temperature gradient and thermal stress inside the concrete, which increases the possibility of cracking, thus reducing the mechanical properties and radiation-proof performance of concrete [11]. Therefore, it is crucial to ensure that radiation-proof concrete still meets the requirements for normal and safe use of the structure after high-temperature deterioration. Compared with traditional heating technology, microwave heating is the use of intermolecular interactions when the microwave incident to the surface of the medium occurs when the transmission and reflection, the medium of the internal intermolecular friction or collision to produce conductivity loss, polarization loss, magnetization loss, and eddy current loss, so as to convert the microwave energy into thermal energy [3]. Some scholars have used microwave heating technology to experimentally study the evolution mechanism of fine microstructure of high-strength concrete and qualitatively determine the high-temperature course of concrete [12]. However, during microwave heating of concrete, the electromagnetic, temperature, and mechanical field evolution cannot be given a quantitative description, and the internal electric field, temperature, stress and strain distributions of concrete cannot be obtained directly from experiments [13]. In recent years, microwave heating process simulation as a research focus and the use of computer technology to assist the microwave heating simulation can effectively reveal the change rule of heat transfer in the microwave heating process, elucidating the microwave heating multi-field coupling problem [14]. Based on the finite element method (FEM), the numerical solution of the microwave heating model can be obtained, and the spatial distribution of the electromagnetic field of the heating process can be visualized and characterized, which provides a basis for the analysis of the processivity of high-temperature deterioration of concrete [15,16,17].

The dielectric loss characteristics exhibited by concrete are related to the chemical composition of the components, such as Al_2_O_3_ and Fe_2_O_3_, and concrete prepared with magnetite as aggregate shows good radiation resistance because of the high percentage of Fe_2_O_3_ content [18]. After microwave irradiation of magnetite stone, it was found that the power of microwave irradiation had a more significant effect on the kinetic properties of magnetite stone compared to the irradiation time [19]. The weakening of the mechanical properties of magnetite ore by microwaves is due to the differences in the sensitivity of different mineral grains to microwaves, which creates temperature stresses within the ore body, eventually leading to rupture by pulling apart internal grain boundaries [20]. However, concrete is a typical porous phase material, and based on the available experimental means, it is difficult to directly monitor the aggregate-mortar interface temperature and internal stress state distribution during microwave heating of concrete [21]. In recent years, microwave heating technology has been simulated and studied by finite element analysis software. Some scholars have established a mathematical model of electromagnetic field and heat transfer to study the mechanism of microwave action on coal rock microcracks, combined with scanning electron microscopy analysis to derive the crack generation mechanism, and proposed a new microwave heating technology for coal bed methane recovery [22]. However, microwave heating of concrete is by no means a simple electromagnetic, thermal problem; at elevated temperatures, mortar, and aggregates exhibit different thermal responses to volumetric deformation. In the research on microwave heating of asphalt concrete, related scholars established a coupled electromagnetic-heat transfer-mechanical model under microwave heating of mortar-aggregate and explored the influence of the dielectric constant and thermal conductivity of the material on the internal heat transfer properties of concrete [23]. Differences in material response led to inhomogeneities in the distribution of electromagnetic and thermal fields within the concrete, generating continuously varying stresses within the concrete and causing cracks to develop [24]. The incorporation of magnetite aggregates significantly enhances the dielectric loss capacity and magnetic properties of concrete [25]. By establishing numerical analysis models, the internal temperature distribution of concrete can be computationally simulated, and the likelihood of segregation between mortar and aggregates due to material-specific differences can be investigated [26,27]. Although experiments have demonstrated that microwaves induce concrete damage [28], systematic studies on microwave heating of magnetite concrete remain scarce. With the growing application of microwave heating technology in concrete repair and recycling, the thermal response of concrete under traditional high-temperature heating methods fails to explain the selective heating characteristics of microwaves. Moreover, research on the thermo-mechanical behavior of concrete containing special aggregates (e.g., magnetite) under microwave fields is notably lacking. In this study, based on an industrial microwave heating system, microwave heating experiments and mechanical loading tests were conducted. For the first time, COMSOL Multiphysics software was employed to simulate both microwave heating and mechanical loading processes in magnetite concrete. Through finite element analysis, the variations in internal electric field distribution, temperature profiles, stress-strain behavior, and residual compressive strength of magnetite concrete were investigated. Experimental and simulation results were cross-validated, confirming the feasibility of the model. By developing a random aggregate model and an electromagnetic-thermal-mechanical multiphysics coupling framework, this study establishes a foundational framework for microwave heating research in concrete, advancing the understanding of microwave-induced damage mechanisms. Furthermore, the analysis of residual compressive strength in magnetite concrete enables more accurate material damage assessment at varying temperatures and supports radiation shielding performance evaluation.

## 2. Materials and Methods

### 2.1. The Material Testing and Specimen Preparation

**Figure 1:**
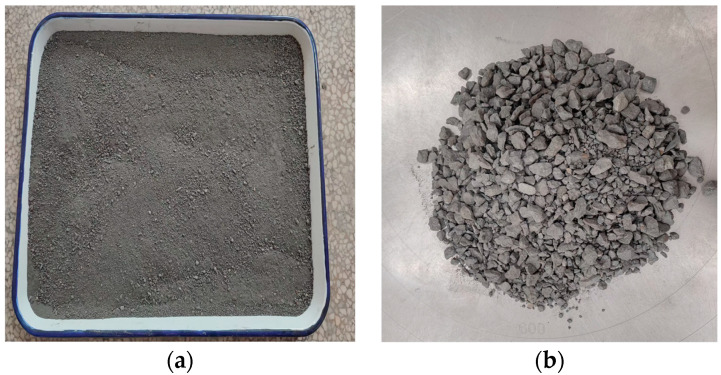
Apparent properties of the material (**a**) cement; (**b**) magnetite.

The experimental cement adopts P∙O 42.5 grade silicate cement, which meets the standard requirements of general silicate cement (GB 175-2023 [29]). Radiation-proof concrete aggregate is selected from magnetite produced in Henan Province. The chemical compositions of cement and magnetite are listed in Table 1, while their apparent characteristics are illustrated in Figure 1.

The sieve test of magnetite coarse and fine aggregates was carried out using the four-part method to obtain magnetite sand with a fineness modulus of 2.55 standard II zone and magnetite stone meeting the grading requirements of nominal particle size 5–20 mm, and the basic properties of magnetite sand and stone are shown in Table 2.

Magnetite ore and magnetite sand of different particle sizes were prepared into coarse and fine aggregates for the experiment according to specific mass proportions. The particle size distribution tables of magnetite sand and magnetite ore are shown in Table 3 and Table 4, respectively.

**Figure 2:**
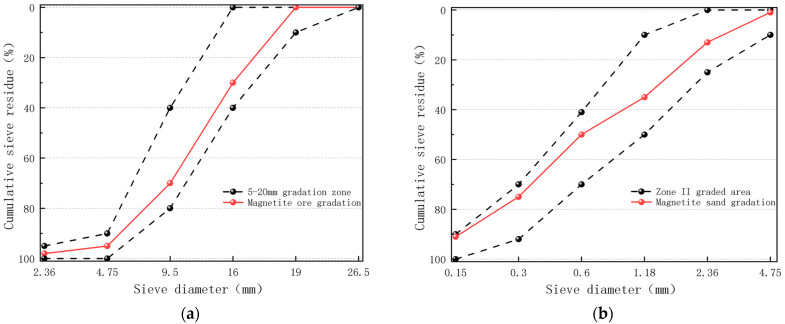
Grading Curve (**a**) Coarse aggregate; (**b**) Fine aggregate.

After data processing, the gradation curves of coarse aggregate and fine aggregate are obtained, as shown in Figure 2. Figure 2 demonstrates that all particle sizes of both coarse and fine aggregates meet the standard requirements specified in “Pebbles and Crushed Stone for Construction” (GB/T 14685-2022 [30]) and “Sand for Construction” (GB/T 14684-2022 [31]), exhibiting excellent gradation characteristics.

Based on the calculation method of the ratio of radiation-proof concrete for iron ore in “Radiation-proof concrete” (GB/T 34008-2017 [32]), the ratio design was carried out, and the magnetite concrete ratio was determined, as shown in Table 5.

**Figure 3:**
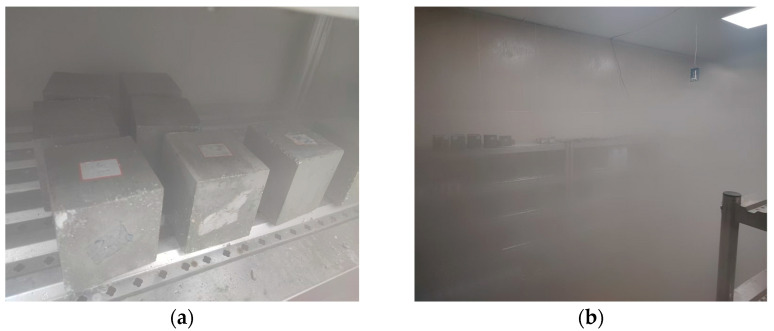
Environmental conservation (**a**) Specimen; (**b**) Conservation room.

A total of 100 cubic specimens (100 mm × 100 mm × 100 mm) were fabricated. The specimens were demolded after 24 h of casting, followed by 28-day standard curing in a controlled chamber. Subsequently, the cured concrete was stored in a dry, ventilated environment for 14 days. The specimens were then dried in a 105 °C constant-temperature forced-air oven for 48 h and finally sealed with plastic wrap after drying. The curing process of the specimens is illustrated in Figure 3.

### 2.2. Experimental Methods

#### 2.2.1. Microwave Heating Experiment

**Figure 4:**
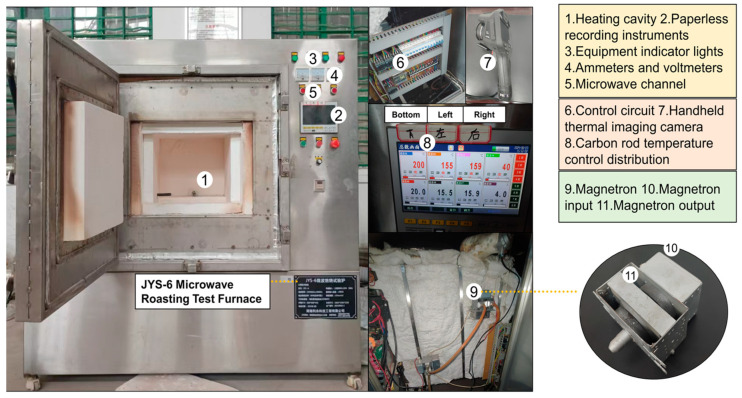
Schematic structure of the JYS-6 microwave roasting test furnace.

The instrument used for microwave heating is the JYS-6 microwave roasting test furnace, which is composed of the control system, water cooling system, magnetron, furnace chamber, and recorder. The surface of the test furnace is made of metal, and the inner wall is laminated with a hollow alumina plate, which can reflect the microwave energy generated by the movement of electrons in the high-frequency electromagnetic field and play a role in heat preservation and insulation. The size of the heating cavity is 400 mm × 480 mm × 340 mm, and there are 6 magnetrons distributed on the outside of the cavity on the left, right, and rear, and the size of the magnetrons is 90 mm × 45 mm × 45 mm, and the emitted microwave frequency is 2450 MHz ± 50 MHz. The microwaves were transmitted into the heating chamber through a waveguide, and the detailed construction of the structure is shown in Figure 4. The concrete specimens are standard cubic blocks with a side length of 100 mm, which are placed in the center of the furnace chamber above the porous alumina plate. Three carbon rods were provided at the lower, left, and rear sides of the cavity to monitor the real-time temperature of the air in the cavity and the surface of the concrete during the heating process and were photographed with a thermal imager at the end of the experiment. The experimental power was uniformly 6 KW, and the concrete was heated from room temperature of 38 °C to 200, 300, 450, 600, and 800 °C, respectively. Each heating interval was heated for a single concrete specimen, and the experiment was repeated three times to avoid chance. When the specimen was heated to the target temperature, the temperature was held constant for 1 h. After heating, the specimen was allowed to cool naturally in the furnace to room temperature and sealed with plastic wrap.

#### 2.2.2. Mechanical Property Pesting Methods

The instrument used for concrete mechanical properties testing is a WAW-EY1000C microcomputer-controlled electro-hydraulic servo universal testing machine, which is loaded according to the Technical Standard for Testing Mechanical Properties of Concrete (GB/T5001-2019) [33]. The loading method was displacement-controlled with a loading rate of 0.01 mm/min to measure the residual concrete strength after microwave heating.

### 2.3. 3D Finite Element Simulation

#### 2.3.1. Stochastic Aggregate Modeling and Microwave Heating Simulation

Concrete specimens have non-uniformity in aggregate placement during fabrication, so a three-dimensional aggregate is randomly generated based on the Monte Carlo method to establish a three-dimensional concrete model containing mortar, aggregate, and interfacial transition zone (ITZ). The process of model creation is as follows:

1. Substrate model creation. The granular aggregate is generated based on the particle size and irregular surface of the aggregate.

2. Graded aggregate generation. Combine Fuller grading curves to determine the aggregate volume percentage for different particle size ranges. Input the minimum spacing between aggregates and create planes for iterative plane contact determination.

3. Interface transition zone generation. The generated graded aggregate is plane cut and reorganized, and the aggregate plane judgment function is invoked to achieve the homogeneity of the interface transition zone.

**Figure 5:**
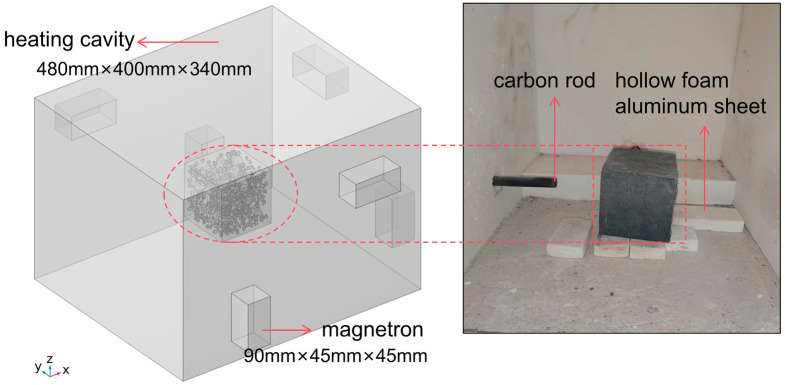
Three-dimensional geometric modeling diagram.

The relevant parameters of the magnetite concrete random aggregate model were set, as shown in Table 6, and 2012 solid units (containing 40,240 surfaces) were generated. The heating chamber and magnetron are simply modeled in COMSOL Multiphysics 6.1, and the generated solid units are imported to obtain the 3D geometric model diagram shown in Figure 5.

#### 2.3.2. Material Parameterization and Basic Assumptions

Concrete can be viewed from a fine-grained point of view as a multiphase material consisting of a cement paste, an aggregate, and an interfacial transition zone (ITZ) at the paste-aggregate interface. When exploring the changes in magnetite concrete during the heating process, ITZ with a thickness of 25 μm was set on the surface of the aggregate, taking into account that the sidewall effect of the aggregate resulted in less cement in its vicinity. The material parameters for the microwave heating setup are shown in Table 7 (microwave frequency of 2.45 GHz). In order to simulate the temperature and stress state distribution between mortar-aggregate under microwave action, the following assumptions are made in the simulation modeling:

(1) The inner cavity of the resonant cavity and the magnetron are conductive materials, and the thickness of the materials is negligible except for the concrete.

(2) The inner walls of the microwave cavity are considered insulating materials, and the heat exchange between the cavity and the external environment during the heating process is neglected. The initial temperature of the concrete before heating is 38 °C.

(3) The size and arrangement of aggregates in the specimen are random. Both the mortar and aggregates are treated as homogeneous materials, and chemical reactions during heating are not considered.

(4) The input power for microwave heating is uniformly set at 6 kW, and the temperature dependence of the material ‘s physical parameters is ignored.

#### 2.3.3. Microwave Heating Principle

Microwave roaster in the microwave heating by the magnetron produces high-frequency electromagnetic waves, concrete specimens through the dielectric loss in the form of absorption of the electromagnetic energy contained. The dielectric constant, conductivity, and magnetic permeability are the main factors in energy loss. In the specimen heating process, the direction of the electric and magnetic fields and the direction of electromagnetic wave motion are perpendicular to each other, the two with spatial variation and the existence of a certain phase difference. For time-varying electromagnetic fields, the time-domain harmonic propagation modes are characterized using a frequency-domain approach and computed by a combination of Maxwell’s equations and Helmholtz vector equations:

(1) $$\nabla \times {\mu_{r}}^{-1}\nabla \times E-{k_{0}}^{2}(\varepsilon_{r}-\frac{j\sigma }{\omega \varepsilon_{0}})E=0$$

where μ_r_ is the relative permeability, E is the electromagnetic intensity (V/m), j is the current density (A/m^2^), σ is the electrical conductivity (S/m), ω is the angular frequency (rad/s), ε_r_ is the relative permittivity (F/m), ε_0_ is the absolute permittivity (F/m), and the spatial wave number k_0_ is denoted as:

(2) $$k_{0}=\frac{2\pi }{\lambda }$$

where λ is the wavelength of the electromagnetic wave (m).

Concrete specimens in the high-frequency electromagnetic field molecules will be polarized and oscillate with the frequency of the electromagnetic field, and molecular friction caused by electromagnetic energy loss will be converted to the internal energy of the medium through the Po Yin Ting theorem can be calculated in the cavity of the electromagnetic loss:

(3) $$p_{eav}=\pi f\varepsilon_{0}\varepsilon_{r}^{\prime \prime }{\left|\vec{E}\right|}^{2}$$

where P_eav_ is the average loss power density (W/m^3^), f is the frequency (Hz), and $\left|\vec{E}\right|$ is the electric field vector (V/m).

In the microwave heating process, electromagnetic energy is converted into heat energy through dielectric loss, magnetic loss, and eddy current loss, resulting in an increase in the internal temperature of the concrete. The depth of microwave penetration into the concrete is δ key parameter that determines the energy distribution, which is calculated as follows:

(4) $$\delta =\frac{c}{2\pi f\sqrt{\varepsilon^{\prime }\cdot \sqrt{1+{\left(\frac{\varepsilon^{\prime \prime }}{\varepsilon^{\prime }}\right)}^{2}}-1}}$$

where c is the speed of light (m/s), and $\varepsilon^{\prime }$ and $\varepsilon^{\prime \prime }$ are the real and imaginary parts of the permittivity, respectively.

Since the electrodeposition loss generated in the concrete specimen cavity is associated with each molecule, each molecule can be considered as a heat source, and this heat transfer relying on the movement of particles within the medium conforms to the heat conduction equation. During the heating process, the temperature, in turn, has an effect on the electromagnetic field distribution, so the energy balance equation is used to analyze the heat transfer process in the furnace cavity:

(5) $$\rho C_{\rho }\frac{\partial T}{\partial t}+\nabla q=Q+Q_{ted}$$

where ρ is the density of the heated object (kg/m^3^), C_ρ_ is the atmospheric heat capacity (J/kg∙K), T is the temperature (K), Q is the density of the heat flux (W/m^3^), Q_ted_ is the microwave heat source (W/m^3^), and q is the heat flux, denoted as:

(6) q=−k∇T

where k is the solid thermal conductivity (W/m∙K).

Concrete is an isotropic material, and the stress-strain of the material under microwave heating satisfies Hooke’s law:

(7) $$S-\left(S_{0}+S_{ext}+S_{q}\right)=C:\left(\varepsilon -\left(\varepsilon_{0}+\varepsilon_{th}+\varepsilon_{hs}+\varepsilon_{pl}+\varepsilon_{cr}\right)\right)$$

where S is stress (Pa), S_0_ is prestress (Pa), S_ext_ is external stress (Pa), S_q_ is viscous stress (Pa), C is the elastic matrix, ε is the elastic strain, ε_0_ is the pre-strain, ε_th_ is the thermal strain, ε_hs_ is the infiltration-expansion strain, ε_pl_ is the plastic strain, and ε_cr_ is the creep.

#### 2.3.4. Boundary Conditions

In the electromagnetic field in the electric field excitation, six rectangular waveguide entrances are defined, including the port boundary, microwave heating cavity, and waveguide, which is defined as the impedance boundary, microwave propagation in the waveguide is affected by the size of the waveguide cross-section and the microwave frequency. The cutoff frequency f_c_ in the model is denoted as:

(8) $$f_{c}=\frac{c}{2}\sqrt{{\left(\frac{m}{a}\right)}^{2}+{\left(\frac{n}{b}\right)}^{2}}$$

where c is the speed of light (m/s), m and n are the microwave modes, and a and b are the waveguide cross-sectional dimensions of 90 mm and 45 mm, respectively. Therefore, the propagation coefficient β at the port boundary is:

(9) $$\beta =\frac{2\pi }{c}\sqrt{f^{2}-f_{c}^{2}}$$

where f is the microwave frequency (Hz).

Since the inner wall of the microwave heating cavity and the waveguide are assumed to be ideal conductors, the impedance boundary condition can be expressed as:

(10) $$\sqrt{\frac{\mu_{0}\mu_{r}}{\varepsilon_{0}\varepsilon_{r}-\frac{j\sigma }{\omega }}}n\times H+E-(n\times E)n=(n\times E_{s})-E_{s}$$

where E_s_ is the electric field vector source (V/m), H is the magnetic field strength (A/m), and μ_0_ is the free space permeability. The concrete surface exchanges heat with the resonant cavity, defining the convective heat transfer boundary:

(11) $$-k\nabla T=h\left(T-T_{air}\right)$$

where h is the convective heat transfer coefficient (W/m^2^·K) at the sample surface, and T_air_ is the room temperature of 38 °C.

##### Grid Division

**Figure 6:**
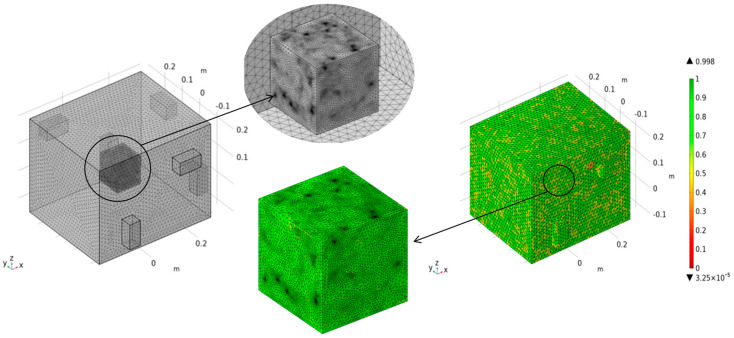
Grid generation and quality evaluation.

In this simulation, customized meshing is used for the furnace chamber, magnetron, and concrete specimens, and the meshing characteristics are shown in Table 8. A total of 48302509 tetrahedral cells were created in the model, and the average cell mass was 0.5403 (as shown in Figure 6), indicating that the model has a good mesh quality evaluation. The relative tolerance of the steady-state solver in the study is 0.001, the maximum number of iterations for the transient solver is 10, the tolerance factor is 1, and the maximum time step is 1 s.

## 3. Results

### 3.1. Model Validation

**Figure 7:**
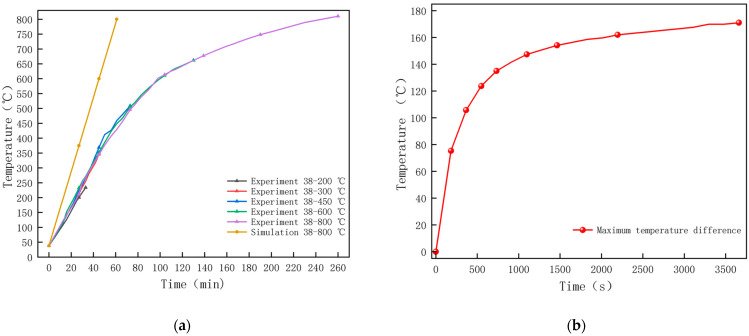
Concrete surface temperature variation: (**a**) Experimental and simulated warming; (**b**) Simulated maximum temperature difference.

Magnetite concrete was repeated three times at different temperatures and the experiment was stopped by monitoring the lower surface temperature of the concrete to the target temperature. In order to determine the validity of the model, collected specimen temperature data were compared with the simulation results for verification, and the variation in concrete surface temperature was obtained, as shown in Figure 7. Figure 7a shows that the concrete warming pattern is partially consistent under microwave heating experiments and simulations. During microwave heating, the simulation results demonstrate that the central temperature at the lower surface of the specimen increases linearly with heating time, exhibiting a heating rate of 13.3 °C/min. In contrast, experimental observations reveal that the temperature at the monitoring point rises approximately linearly with heating time at lower temperatures, with a measured heating rate of 8.3 °C/min. This discrepancy is primarily attributed to heat loss in the microwave-roasting furnace during the heating process, which explains why the simulated heating rate of the concrete specimen exceeds the experimental values. Microwave heating experimental data further indicate that when the temperature exceeds 600 °C, the heating rate gradually slows. This phenomenon is mainly caused by continuous changes in the dielectric constants of the mortar and aggregates during heating. The heterogeneity of concrete, combined with these variations in dielectric properties, results in non-uniform heating behavior, thereby influencing the heating rate. As the heating duration extends, energy generation and loss gradually equilibrate, ultimately reaching a dynamic equilibrium state. In subsequent sections, we will provide a detailed discussion of the configuration of material parameters. It can be seen in Figure 7b that when heated for up to 60 min, the maximum temperature difference on the surface is 171 °C.

Due to the different material properties of mortar and aggregate, the concrete is subjected to different thermal states at various locations, and the apparent characteristics, thermal imaging, and simulation results that occur when the concrete is heated to different temperatures are given in Table 9. The results show that the simulation results are in good agreement with the thermographic images, and there is inhomogeneity in the temperature distribution on the concrete surface. On the same plane of magnetite concrete (e.g., plane abcd), “hot zones” are formed at some edge locations (e.g., zones a and c), and “cold zones” are formed at some edge locations (e.g., zones d and e). The temperatures in the center regions of the planes abcd and cdef are lower than the temperatures in the other regions, and this phenomenon is most pronounced at 200 °C and 300 °C. The experimental phenomenon shows that the cracks are generated from the edge and gradually expand inward, which is consistent with the direction of heat transfer from the high-temperature region to the low-temperature region, which suggests that there is a certain correlation between the temperature and the mechanical field.

**Figure 8:**
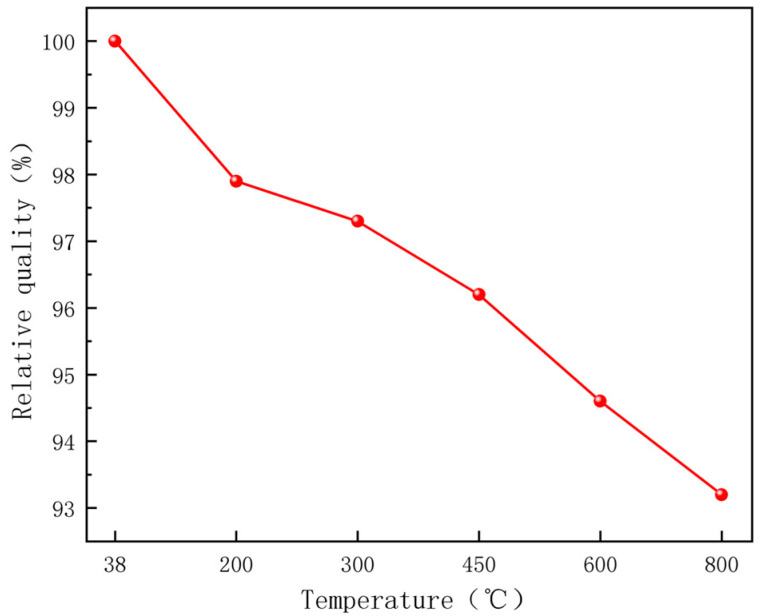
Relative mass of magnetite concrete after high-temperature treatment.

During microwave heating, concrete undergoes a series of physical and chemical reactions at elevated temperatures, leading to corresponding changes in mass. Based on the mass variations in concrete under high-temperature exposure, the degradation degree and compositional alterations of concrete can be indirectly assessed. When heated to 200 °C, 300 °C, 450 °C, 600 °C, and 800 °C, the measured mass of the specimens was only 97.9%, 97.3%, 96.27%, 94.6%, and 93.2% of their initial mass before the experiment, respectively. Figure 8 illustrates the relative mass of magnetite concrete after high-temperature treatment. The results indicate that the mass of magnetite concrete continuously decreases with increasing temperature, accompanied by a gradual color transition of the specimen surface from grayish-green to pale white and then to light red. At heating temperatures of 200 °C and 300 °C, surface-free water evaporates, resulting in the emergence of small bubble pores and initial microcracks, while the specimen color gradually whitens. At 450 °C, the hydration product Ca(OH)_2_ begins to decompose, microcracks proliferate in both number and dimensions (length and width), and the specimen edges exhibit slight reddish discoloration. By 600 °C, hydration products are extensively decomposed, the cement paste becomes friable, cracks further propagate to form penetrating fractures, and reddish hues appear uniformly across the surface. At 800 °C, CaCO_3_ decomposition occurs, crack widths deepen, extensive surface cracking develops, edge spalling is observed, localized fissures emerge, and the overall surface adopts a light red coloration.

### 3.2. Electromagnetic Thermal Effects

**Figure 9:**
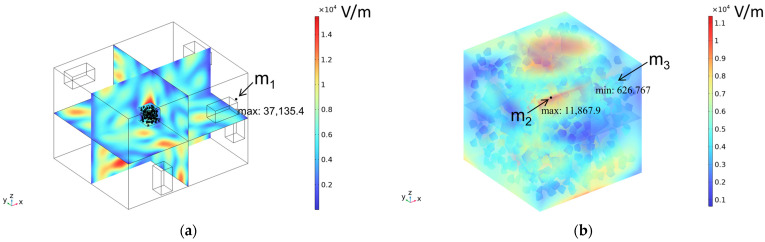
Electric field strength distribution (**a**) Heating chamber (**b**) Concrete.

The electric field distribution is usually related to the microwave frequency, the waveguide size, the size of the concrete, the dielectric properties, and the position in the heating cavity; the waveguide cutoff frequency is 1.67 GHz because of the resonance cavity is set as an impedance boundary condition, the microwave in the resonance cavity is constantly reflected to form a standing wave field, which generates a strong and weak electric field, and the distribution of the electric field strength inside the heating cavity and the concrete is shown in Figure 9. Table 10 reflects the distribution of electric field strength and electromagnetic power loss on the surface and cross-section of the concrete; the maximum electric field strength m_1_ in the cavity is 37,135.4 V/m, and when the microwave passes through the concrete, the electric field strength appears to decay, the maximum electric field strength m_2_ on the surface of the specimen body is 11,867.9 V/m, and the minimum electric field strength m_3_ is 626.767 V/m. Under the action of high-frequency electromagnetic fields, the polarity orientation of polar molecules in concrete continuously changes, causing molecular motion and friction effects, thereby converting microwave energy into molecular kinetic energy and internal energy. This energy conversion is represented by electromagnetic power loss. From Table 10, it can be seen that the electric field strength and electromagnetic power loss of concrete are consistent in surface and cross-sectional distribution.

**Figure 10:**
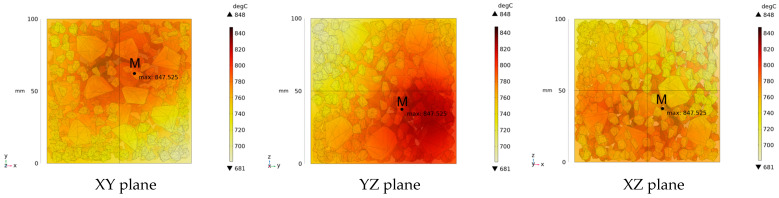
Three-dimensional view of the highest temperature position of concrete.

Due to the non-uniform distribution of materials and differences in dielectric loss, aggregate regions exhibit stronger energy absorption owing to their higher dielectric constant and electromagnetic loss. As shown in Figure 10, the location of the maximum temperature within the concrete under microwave heating is identified at point M (60.2,62.8,37.6). For clarity in representation, point f is set as the origin of the spatial coordinate system (0,0,0). In this paper, based on the calculation of the electromagnetic field distribution, the penetration depth of microwaves in concrete is approximately 30–40 mm, which corresponds to the location of the highest temperature.

**Figure 11:**
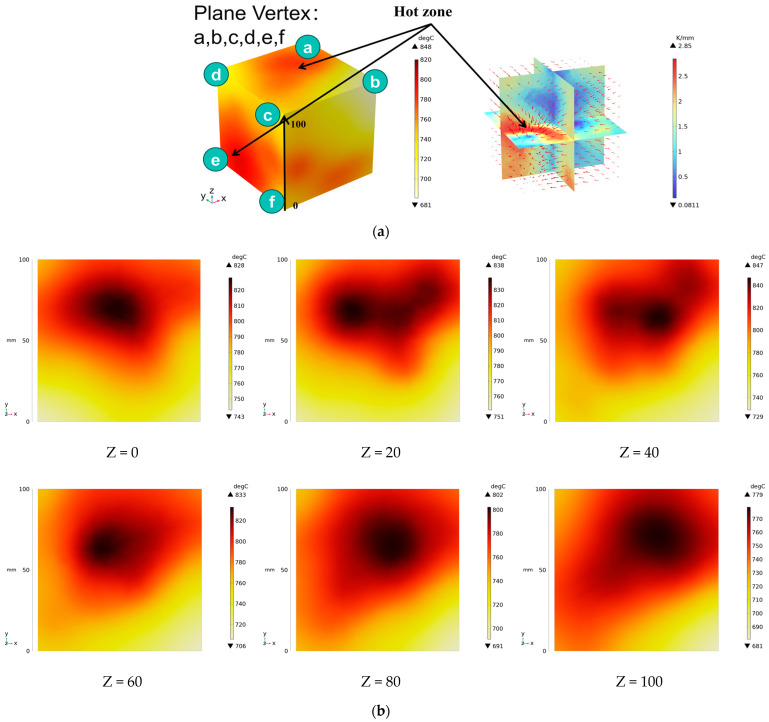
Temperature distribution: (**a**) Body surface and internal; (**b**) Change with Z-axis depth.

Figure 11 presents the temperature distribution on the surface and within the specimen at a heating time of t = 2909 s. As shown in Figure 11a, the temperature across the concrete surface and interior exhibits non-uniform distribution, with “hot zones” observed at the edge regions (zones a and e) and “cold zones” at the corner regions (zones b and d). This phenomenon is caused by uneven microwave distribution on the concrete surface, where higher energy absorption occurs in regions with stronger electric fields, resulting in elevated temperatures. Figure 11b displays the cross-sectional temperature distribution profile along edge fc (i.e., the z-axis) at 20-mm intervals. It can be observed that the temperature difference between the surfaces at z = 0 mm and z = 100 mm is smaller than the temperature gradient within the internal planes. This is due to the characteristic of dielectric loss of materials, which causes concrete to be in a “heat storage” state, with internal temperature higher than external temperature, resulting in the generation of plane temperature difference.

**Figure 12:**
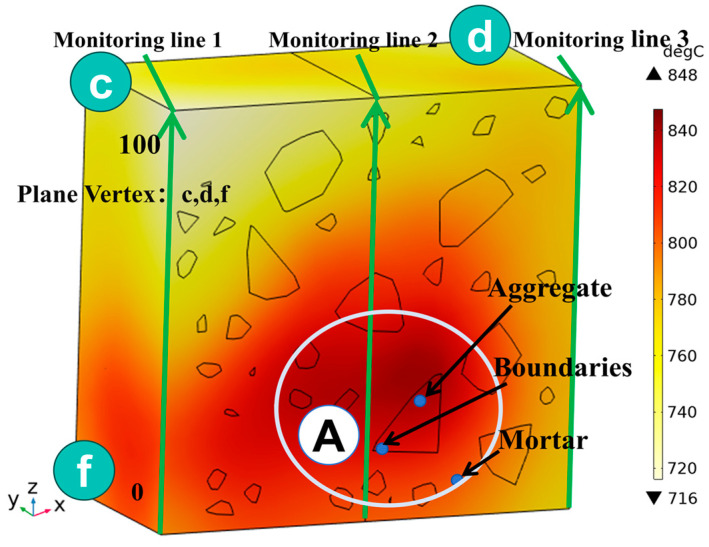
Temperature monitoring line and boundary point probe distribution.

**Figure 13:**
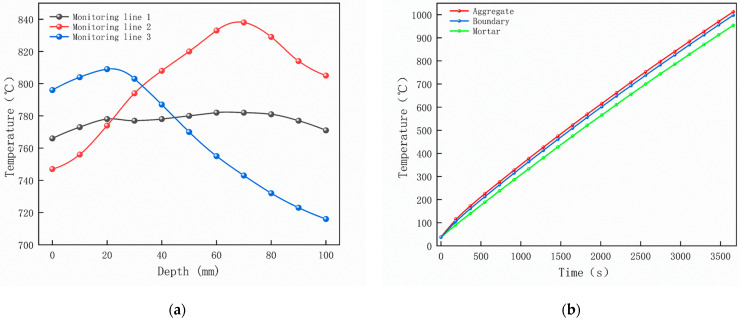
(**a**) Monitoring line temperature with depth; (**b**) Aggregate, boundary, and mortar temperatures with time.

Figure 12 shows that boundary point probes were set up in the mid-section of the specimen (y = 50 mm) in the aggregate, mortar, and interface transition zone (hereafter referred to as the boundary), respectively, and three temperature monitoring lines were set up along the positive direction of the z-axis at (0, 50, 0), (50, 50, 0), and (100, 50, 0), ranging from 0 mm to 100 mm. In order to clearly compare the variation in temperature along the depth of the specimen and the effect of the material on the temperature in the same region (region of A), the temperature distribution with depth and the material temperature with time at different locations of the specimen are shown in Figure 13a,b. The results show that the concrete temperature distribution exhibits a regional pattern, with temperatures detected on monitoring lines 1, 2, and 3 increasing and then decreasing as the z-value increases from 0 mm to 100 mm. In the region of Figure 13a, the warming rate of Aggregate, Boundary, and Mortar in magnetite concrete heated from room temperature to 800 °C gradually decreases from 0.426 °C/s, 0.295 °C/s, and 0.377 °C/s to 0.23 °C/s, 0.224 °C/s, and 0.23 °C/s, respectively. This is caused by the inherent thermal conductivity of each phase of concrete and the limited amount of heat transferred by heat conduction during warming makes the temperature distribution of the specimen uneven.

### 3.3. Thermal Stress Evolution

The temperature difference between the phases of concrete grows with the heating time because of the deformation constraints of the specimen and the differences in the thermal expansivity of the mortar-aggregate. Uneven distribution of temperature produces uneven expansion inside the concrete, cracks inside and on the surface of the specimen, and stress concentration at the interface transition zone, and the stress-strain evolution inside the concrete is shown in Table 11. From the table, it can be seen that the internal stress and strain distributions of the specimens are also non-uniform, both of which show that the interior is larger than the surface, which is similar to the discussion of the surface and internal temperature distributions of concrete in the previous section. On the same facet, high stresses and strains are concentrated in the interfacial transition zone.

**Figure 14:**
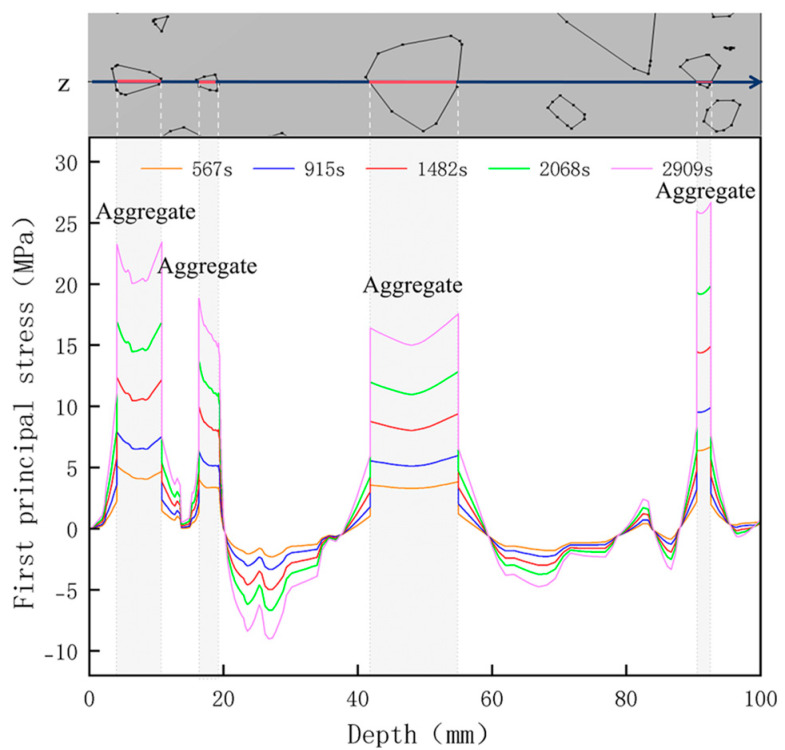
Distribution of the first principal strains along the z-axis at different temperatures.

Figure 14 shows the change in the first principal stress during the growth from 0 mm to 100 mm on monitoring line 2 at different temperatures. When the concrete was heated to 200, 300, 450, 600, and 800 °C, the heating time was 567 s, 915 s, 1482 s, 2068 s, and 2909 s, respectively, and the maximum tensile stress at the aggregate on the monitoring line 2 reached 6.69 MPa, 9.92 MPa, 14.93 MPa, 19.89 Mpa, and 27.37 MPa, respectively, and the average rise rate was 3.3 × 10^−2^ MPa/°C; The maximum compressive stress at the mortar on monitoring line 2 reached 2.07 MPa, 3.05 MPa, 4.61 MPa, 6.2 MPa, and 8.39 MPa, respectively, with an average rate of increase of 2.9 × 10^−3^ MPa/℃. It can be seen that tensile stresses are exhibited at the aggregate and boundaries and compressive stresses at the mortar. The maximum tensile stress occurs at the boundary, and stress concentration occurs.

**Figure 15:**
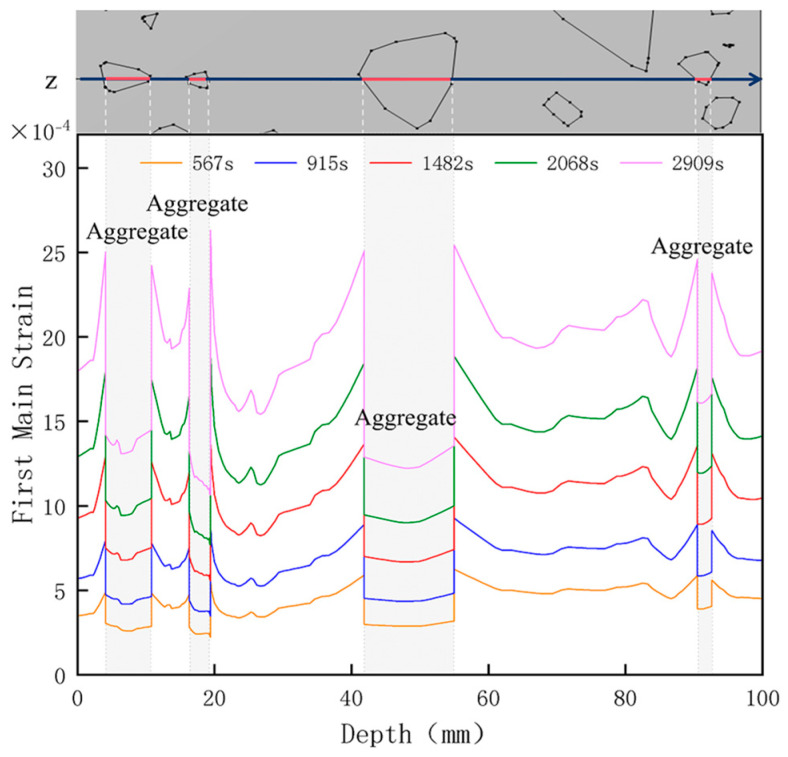
Distribution of the first principal stress along the z-axis at different temperatures.

Figure 15 shows the change in the first principal strain during the growth from 0 mm to 100 mm on monitoring line 2 at different temperatures. It can be seen that there is some similarity between the first principal strain distribution and the first principal stress distribution, with strain concentrations occurring at the boundaries, but the strains generated at the aggregate are smaller than those at the mortar. During microwave heating, the maximum first principal strain increased from 2.95 × 10^−4^/°C to 3.3 × 10^−4^/°C at the mortar and from 1.4 × 10^−4^/°C to 1.5 × 10^−4^/°C at the aggregate. When heated to 800 °C, the maximum strain at the mortar was 2.68 × 10^−3^, and the maximum strain at the aggregate was 1.69 × 10^−3^, at which time the maximum strain at the mortar was 1.59 times the maximum strain at the aggregate. Magnetite concrete mortar has a larger coefficient of thermal expansion and smaller modulus of elasticity compared to aggregate, so it produces higher deformation in the mortar than in the aggregate. The stresses at aggregate, boundary, and mortar increase with increasing strain.

### 3.4. Compressive Strength Analysis

**Figure 16:**
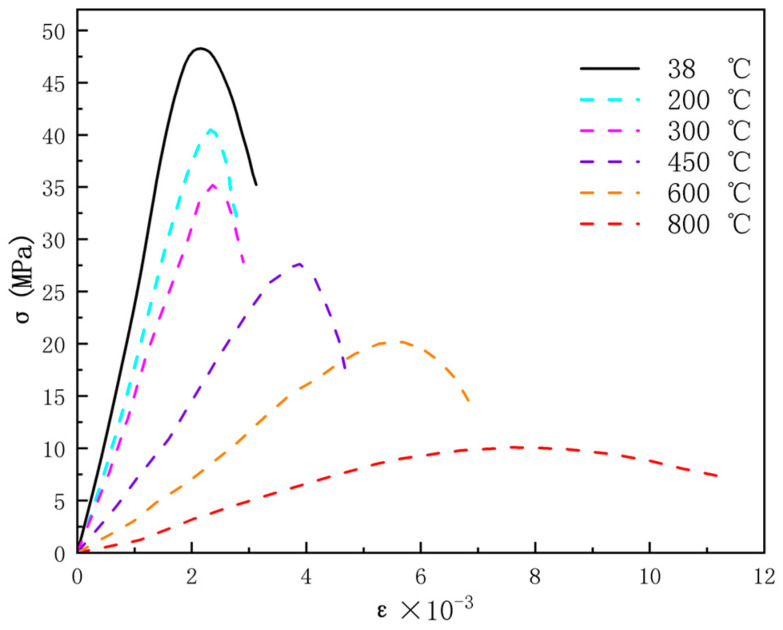
The axial compressive stress-strain curve of magnetite concrete after exposure to high temperature.

In the process of microwave heating, the stress at the aggregate, boundary, and mortar increases with an increase in strain. A displacement load of 0.01 mm/min was applied at the top surface (z = 0 mm), and the experimental results showed that the standard cube compressive strength of magnetite concrete at 38 °C is 47.4 MPa. The axial stress-strain curve of magnetite concrete after exposure to high temperature is illustrated in Figure 16. From the graph, it can be observed that the stress-strain curve of magnetite concrete after exposure to high temperatures still exhibits an elastic stage, a plastic stage, and a failure stage. As the heating temperature increases, both the peak value of the stress-strain curve (axial compressive strength) and the slope of the ascending segment (elastic modulus) of magnetite concrete decrease. When the temperature exceeds 450 °C, the peak point gradually shifts to the right, and the descending segment of the curve becomes more gradual, indicating an increase in the ductility of the concrete. At 800 °C, the stress-strain curve tends to flatten, and the load-bearing capacity significantly decreases.

To investigate the effect of temperature on the residual compressive strength of magnetite concrete, six different temperatures were selected, and three experiments were conducted at each temperature. Experimental data are presented in Table 12.

The number of factor levels is six, with three experiments conducted under each level, resulting in a total of 18 experiments. To study the extent of the influence of temperature on the results, an F-test was performed. The relevant calculation results are listed in the Analysis of Variance (ANOVA) Table in Table 13. From the F-distribution table, it is found that F > F_0.05_ (5,12) = 3.11 and F > F_0.01_ (5,12) = 5.06, indicating that temperature has a highly significant effect on the residual compressive strength.

**Figure 17:**
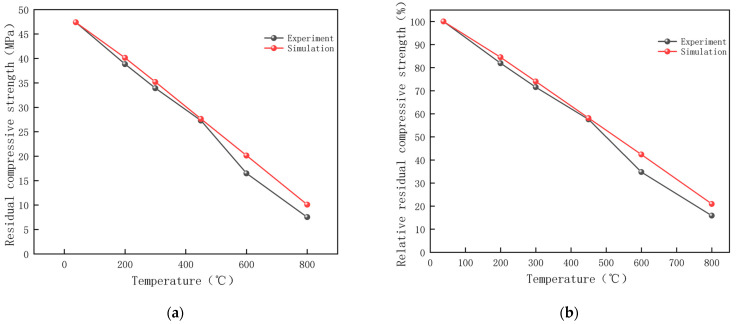
Change in the strength of magnetite concrete under microwave heating (**a**) Residual compressive strength (**b**) Relative residual compressive strength.

The variation in the strength of magnetite concrete measured by experiments and simulations is shown in Table 14. In order to study the deterioration of magnetite concrete at different temperatures more intuitively, the index of relative residual compressive strength is introduced. The relative residual compressive strength is the ratio of the residual compressive strength at different temperatures to the residual compressive strength at 38 °C. The residual compressive strength and relative residual compressive strength of magnetite concrete after high-temperature action under test and simulation were obtained by data normalization, as shown in Figure 17.

Due to the heterogeneous nature of the material and the random propagation paths of the cracks differing from the idealized assumptions made in the simulation, there is a deviation in the residual compressive strength of the magnetite concrete measured in experiments and simulations at different temperatures, with a maximum deviation of only 7.58%. A comprehensive comparison revealed that the residual compressive strength of concrete specimens under microwave heating decreased with an increase in heating temperature. The average rates of decrease in residual compressive strength of magnetite concrete calculated experimentally and by simulation were 0.053 MPa/°C and 0.049 MPa/°C, respectively. When the temperature is 450–600 °C, the residual compressive strength of magnetite concrete decreases to below 60%, and the largest decrease (0.07 MPa/°C) is the largest, and the mechanical properties of concrete deteriorate most seriously at this time, accompanied by penetrating cracks. This is due to the fact that at high temperatures, the internal pore pressure and component changes in concrete increase crack development, causing concrete damage and reducing strength properties [37]. Due to the strong microwave absorption capacity of the aggregate during heating, the internal heating rate of the specimen during the heating process is greater than the external air convection heat transfer, resulting in the internal and external temperature difference and non-uniform thermal expansion to increase the stress generation, accelerate the crack development, resulting in persistent damage to make the strength decrease [38,39].

## 4. Discussion

### 4.1. Analysis of Study Results and Limitations

**Figure 18:**
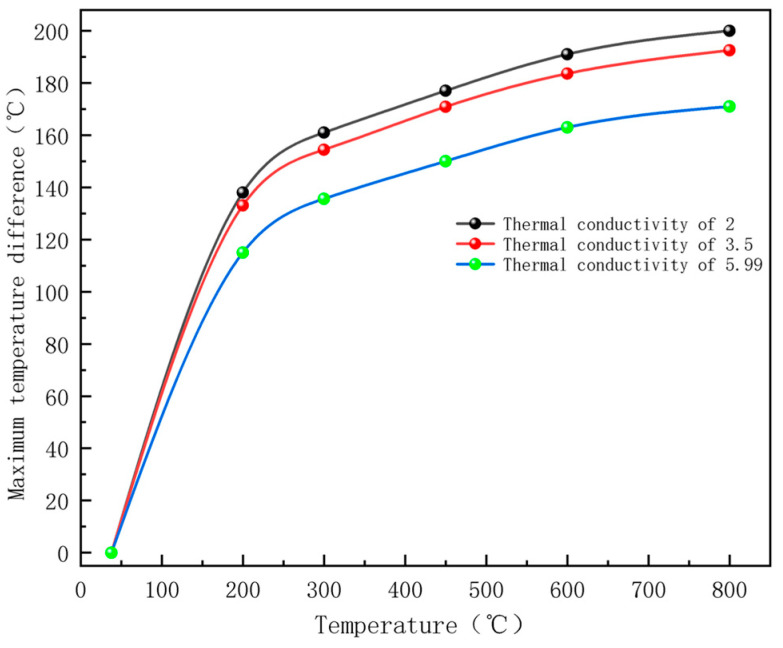
The temperature difference between the inside and outside of magnetite concrete in microwave heating.

This study employs COMSOL-based numerical simulations to investigate the thermo-mechanical response of magnetite concrete under microwave heating, establishing a foundational framework and theoretical model for microwave heating research on concrete. During the simulation, due to a scarcity of experimental data on dielectric constants, thermal conductivity, and other parameters of magnetite concrete at temperatures above 300 °C, as well as limitations in experimental conditions, the temperature dependence of material properties was neglected in parameter settings. While this simplification does not alter the overall heating pattern of the specimen, it may introduce localized temperature calculation errors at specific heating stages. The finding that the internal temperature of the specimen exceeds the surface temperature under microwave heating aligns with previous simulation results for concrete [24]. Using the random aggregate model developed in this study, predictions of the thermal response of concrete at different temperatures can be made by adjusting relevant material parameters, thereby assessing the mechanical performance degradation of the specimen. When heated from room temperature to 800 °C, the primary component of magnetite (Fe_3_O_4_) transforms into Fe_2_O_3_, reducing thermal conductivity to approximately 2 W/(m·K) [36]. The simulated maximum temperature difference between the interior and surface of the concrete during microwave heating is shown in Figure 18. As can be seen from Figure 18, with an increase in temperature, the thermal conductivity of magnetite decreases, and the temperature difference between the inside and outside of the final specimen gradually increases. When the thermal conductivity of magnetite decreases to 2, and the concrete is heated to 800 °C, the temperature difference between the inside and outside exceeds 200 °C.

Concrete, as an anisotropic material, exhibits microwave absorption characteristics that are influenced by the arrangement and proportion of aggregates. However, in the actual preparation of concrete specimens, it is challenging to control the distribution of aggregates. Therefore, we assumed that the mortar and aggregates are homogeneous and continuous, and after determining the optimal mix ratio, we created a random aggregate model for simulation. Under this assumption, the localized temperature within the concrete may be lower than the actual value, affecting the stress-strain response at the interfacial transition zone (ITZ) and potentially overestimating the overall strength of the concrete. Under the condition of not changing the volume ratio of each particle size, only changing the random generation position of the aggregate and the number of irregular surfaces, the results show that the residual compressive strength after microwave heating has no significant change.

In the simulation of the microwave heating equipment (roasting furnace), the outer surface of the cavity is wrapped with multi-layer thermal insulation blankets (as shown in Figure 4). To simplify the calculations, the actual heat exchange between the cavity and the external environment during heating was neglected, and the inner walls of the microwave cavity were assumed to be thermally insulating materials [26,28,40]. This assumption underestimates the heat loss of the cavity, causing the temperature of the concrete to rise faster than in reality during prolonged heating. Although this may affect the accuracy of the heat transfer simulation, the thermal-mechanical response of the concrete at different heating temperatures will not significantly change.

As the heating temperature of the concrete gradually increases, a series of physicochemical reactions (such as changes in water content and decomposition of hydration products) occur. These reactions may alter material properties, thereby influencing the initiation and propagation of cracks under internal stress. Since incorporating the chemical changes in concrete during heating into the model would significantly increase computational complexity, this study indirectly assessed the phase composition changes in concrete through experiments by measuring the mass loss of specimens after microwave heating and combining it with macroscopic surface characteristics.

### 4.2. Research Contributions and Application Discussions

In traditional concrete modeling, aggregates are often simplified as spherical shapes to reduce computational complexity. However, using randomly shaped aggregates better reflects the actual preparation process of concrete, thereby enhancing the credibility of the results. This study focuses on magnetite concrete and establishes a multi-field coupling model integrating electromagnetic, thermal, and mechanical fields. Analyzing the residual compressive strength of concrete at different temperatures provides a foundational framework for research on microwave heating of concrete, contributing to the understanding of microwave-induced damage mechanisms. The simulation approach identifies the location of the maximum temperature under microwave heating, which helps validate theoretical studies on microwave penetration depth in concrete.

In the previous section, the influence of aggregate thermal conductivity on the thermal response demonstrates that the model is not limited to magnetite concrete. By adjusting relevant material parameters, the model can be extended to other types of concrete, enabling rapid prediction of their thermal-mechanical responses under microwave exposure. The maximum stress is located in the interfacial transition zone (ITZ), where cracks initiate and propagate first, consistent with previous research findings [28]. Building on this, this study conducts mechanical loading simulations and validates the results through experiments, enabling damage assessment of material properties at different temperatures.

When magnetite concrete is exposed to temperatures between 450 °C and 600 °C, its mechanical properties deteriorate rapidly, with the residual compressive strength dropping to less than 60% of its initial value. Enhancing the concrete’s ability to absorb microwaves or improving the interfacial bonding strength can improve its mechanical performance under electromagnetic fields. Material optimization, such as incorporating nano-SiO_2_ or TiO_2_, can alter the morphology of cement hydration products, resulting in a denser cement paste and higher compressive strength [41]. Within the 450–600 °C range, the strength loss rate of magnetite concrete reaches 0.07 MPa/°C. Research has shown that when the residual compressive strength of concrete decreases to about 60%, the linear attenuation coefficient of 100 mm thick concrete decreases by 10.16% [42]. Based on the research results of this paper, the residual compressive strength is used as the maintenance trigger index, and the state of the radiation shielding structure can be evaluated through regular nondestructive testing. The temperature and strain field can be monitored in real-time by embedding fiber grating sensors in concrete, which can realize the health monitoring of concrete. Microwave selective heating characteristics can also be used to irradiate the crack area locally so that the mortar at the crack can be re-sintered to achieve the purpose of local crack repair.

Beyond the assumptions discussed above, real-world service environments for radiation-shielding concrete may involve unstable radiation heat sources, long-term cyclic radiation exposure, and wet-dry cycles. Therefore, future research will focus on model development to explore the thermal-mechanical responses of concrete under different microwave powers and frequencies, variations in moisture content during heating, and cyclic heating and cooling processes. Additionally, experiments will be conducted to refine material parameters and enhance the reliability of the conclusions.

## 5. Conclusions

In this paper, the simulation of microwave heating of magnetite concrete under coupled electromagnetic, temperature, and mechanical fields is realized by establishing a numerical model of random aggregate. The thermal stress evolution law of magnetite concrete was studied in detail, and the changes in residual compressive strength and relative residual compressive strength of magnetite concrete after microwave heating were analyzed and mutually verified by experiments, and the following conclusions were obtained:

1. The distribution of electric field strength and electromagnetic power loss on the surface of a magnetite concrete body under microwave heating is basically the same. When the microwave passes through the concrete, the electric field strength is attenuated, and the strong electric field inside the concrete is mostly concentrated at the aggregate, and the lowest field strength is at the interface transition zone.

2. The internal temperature of magnetite concrete under the microwave heating method is higher than the surface temperature and is accompanied by obvious surface characteristics during the heating process. The temperature difference between the inside and outside of the concrete body surface continues to rise during the heating process, with the maximum temperature difference exceeding 150 °C at 800 °C. The thermal conductivity of the material affects the temperature transfer rate, and the thermal conductivity of the aggregate is negatively correlated with the temperature difference between the inside and outside. The surface color of the concrete changed from grayish green to light white to light red as the heating temperature increased, and the number of surface cracks and crack width increased. Cracks interconnect and form penetrating cracks at 450–600 °C, with spalling and cracking occurring at 800 °C and above. The rate of warming in the aggregate, mortar, and interfacial transition zones decreases gradually with increasing heating time.

3. During microwave heating of magnetite concrete, the maximum values of the first principal stress and the first principal strain occur in the interface transition zone. The stresses generated at the magnetite concrete aggregate are higher than those at the mortar, and tensile stress concentration occurs at the interfacial transition zone, with the maximum tensile stress increasing at an average rate of 3.3 × 10^−2^ MPa/°C. The compressive stress was mainly distributed at the mortar, and the average growth rate of the maximum compressive stress was 2.9 × 10^−3^ MPa/°C. The rate of strain and strain growth at the concrete mortar was greater than at the aggregate, with the maximum strain at the mortar at 800 °C being 1.59 times the maximum strain at the aggregate.

4. The residual compressive strength of concrete specimens under microwave heating decreases with increasing heating temperature. The effect of temperature on the residual compressive strength of magnetite concrete is very significant. The residual compressive strength of magnetite concrete calculated by simulation fits the actual situation with a maximum difference in strength values of only 7.58%. At 450 °C, the residual compressive strength of magnetite concrete decreases to less than 60% of its initial value. When the temperature is in the range of 450–600 °C, the relative residual compressive strength of magnetite concrete exhibits the most significant decline (0.07 MPa/°C), indicating the fastest deterioration of its mechanical properties. Real-time monitoring of temperature and strain fields in concrete through methods such as embedding sensors can be utilized for the health monitoring of concrete structures.

## Figures and Tables

**Table 1:** Chemical composition of cement and magnetite (%).

Typology	CaO	SiO_2_	Fe_2_O_3_	Al_2_O_3_	MgO	K_2_O	Na_2_O	SO_3_
Cement	62.05	22.51	3.30	4.33	1.49	1.28	0.96	2.02
Magnetite	3.26	7.94	78.52	5.55	2.43	0.11	0.54	0.26

**Table 2:** Basic properties of aggregates.

Typology	Particle Size (mm)	Apparent Density (kg/m^3^)	Moisture Content (%)	Water Absorption (%)	Crushing Value Index (%)
Magnetite ore	5–20	4415	0.1	0.3	7.1
Magnetite sand	0–5	4545	0.1	0.7	—

**Table 3:** Magnetite ore particle gradation.

Mesh Diameter (mm)	Sieve Residue Measurement (%)	Cumulative Sieve Residue (%)	Cumulative Screening Range (%)
19	0	0	0–10
16	30	30	0–40
9.5	40	70	40–80
4.75	25	95	90–100
2.36	3	98	95–100

**Table 4:** Magnetite sand particle gradation.

Mesh Diameter (mm)	Sieve Residue Measurement (%)	Cumulative Sieve Residue (%)	Cumulative Screening Range (%)
4.75	1	1	0–10
2.36	12	13	0–25
1.18	22	35	10–50
0.6	15	50	41–70
0.3	25	75	70–92
0.15	16	91	90–100

**Table 5:** Magnetite concrete mix ratios.

Strength Class	Water-Cement Ratio	Volumetric (kg/m^3^)			
		Coarse Aggregate	Fine Aggregate	Cement	Water
C30	0.51	1941.8	1140.4	362.7	185

**Table 6:** Aggregate parameters.

Size (mm^3^)	Number of Aggregate Vertices	Aggregate Radius (mm)	Volume Fraction (%)	ITZ Thickness (mm)	Minimum Spacing of Aggregates (mm)
100 × 100 × 100	12	0.1–0.3	13	0.024	0.01
		0.3–2.5	12		
		2.5–4.5	25		
		4.5–10	18		

**Table 7:** Material parameters.

Physical Properties	Air ^1^	Mortar	Magnetite
Density (kg/m^3^)	1.29	2300	4480
Relative dielectric constant	1	6.91–0.753j ^2^	9.5–2.38j ^3^
Relative magnetic permeability	1	1	1
Thermal conductivity	-	0.98 ^2^	10 ^4^
Electrical conductivity (S/m)	0	0.98	0
Coefficient of thermal expansion (10^−6^/K)	-	12 ^2^	5.99 ^5^
Modulus of elasticity (10^9^Pa)	-	25	130
Poisson’s ratio	-	0.2	0.26

^1^ Taken from COMSOL 6.1 built-in material library. ^2^ Taken from Ref [27]. ^3^ Taken from Ref [34]. ^4^ Taken from Ref [35]. ^5^ Taken from Ref [36].

**Table 8:** Mesh characteristics of the model.

Unit Parameters	Furnace Chamber and Magnetron	Concrete
Maximum cell size (mm)	24.47	2.31
Minimum cell size (mm)	7.342	0.279
Maximum cell growth rate	1.35	1.35
Curvature factor	0.6	0.6
Narrow area resolution	0.5	0.5

**Table 9:** Apparent properties of concrete at various temperature thresholds.

Temperature (°C)	Experimental Appearance	Thermal Imaging	Simulation
38	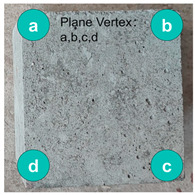	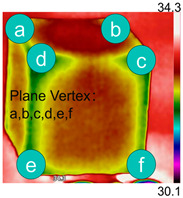	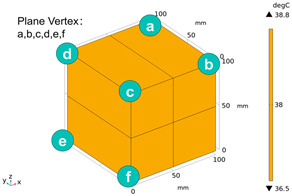
200	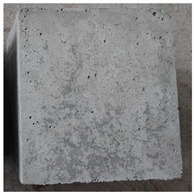	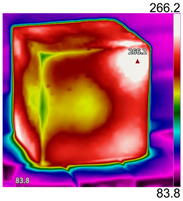	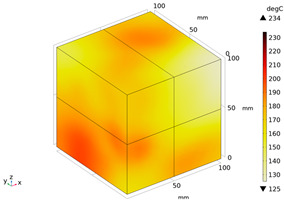
300	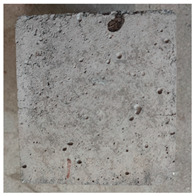	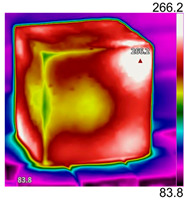	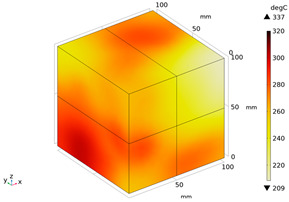
450	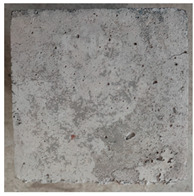	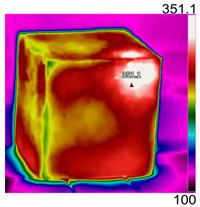	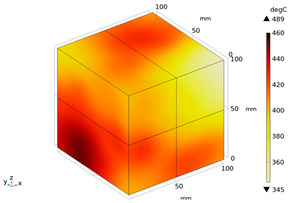
600	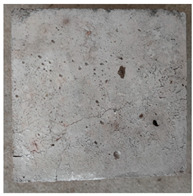	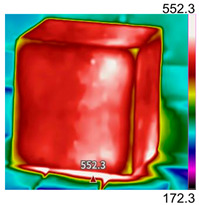	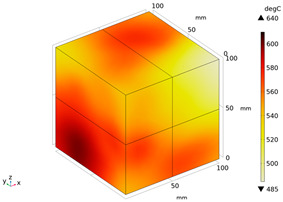
800	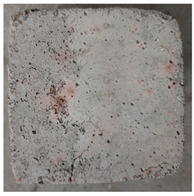	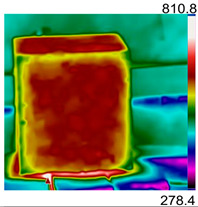	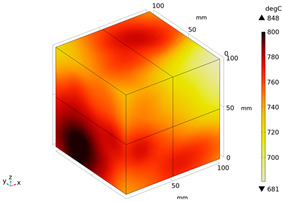

**Table 10:** Concrete surface and cross-section electric field strength and electromagnetic losses.

Electromagnetic Distribution	Electric Field Strength (V/m)	Electromagnetic Power Loss (W/m^3^)
Body surface	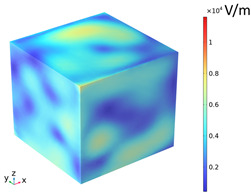	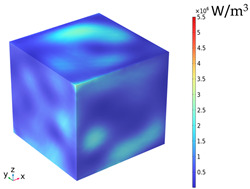
XY plane (z = 50 mm)	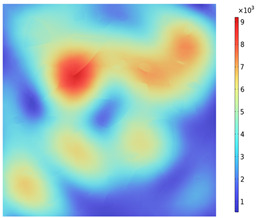	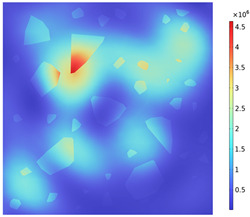
YZ plane (x = 50 mm)	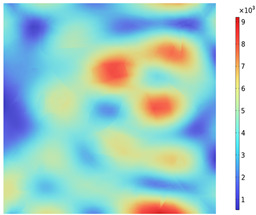	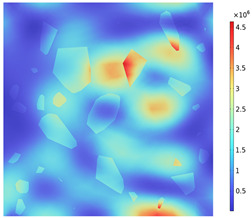
ZX plane (y = 50 mm)	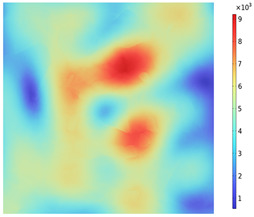	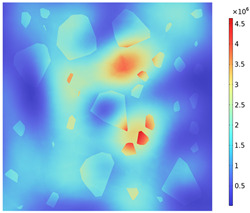

**Table 11:** Internal stress-strain evolution of concrete.

Depth Along z-Axis (mm)	Stress Distribution	Strain Distribution
0	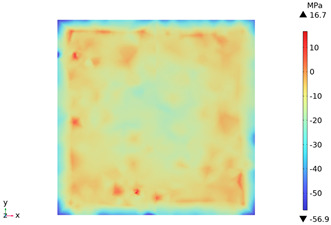	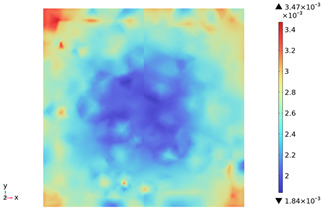
20	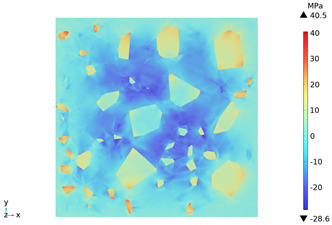	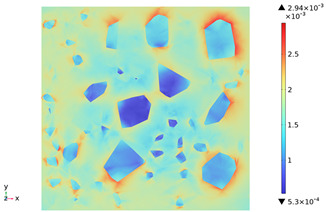
40	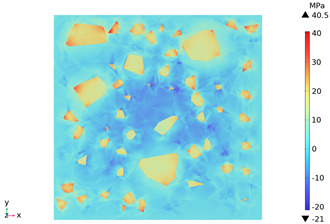	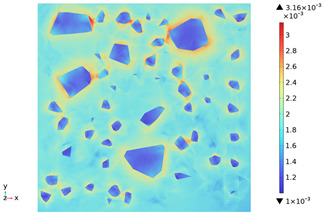
60	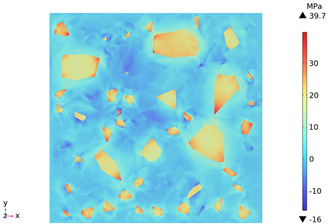	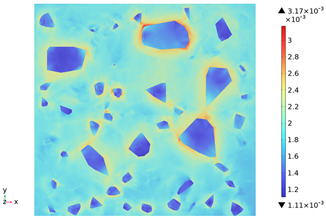
80	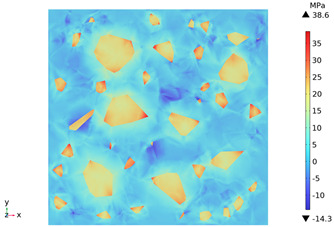	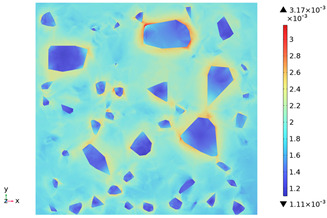
100	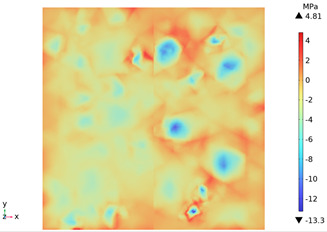	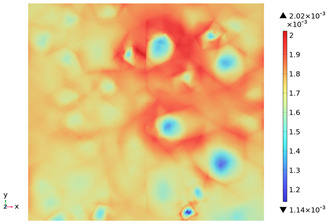

**Table 12:** Experimental Results of Residual Compressive Strength of Concrete under Microwave Heating.

Temperature (°C)	Residual Compressive Strength (MPa)			Averaged Within the Group (MPa)
38	47.5	48.25	46.45	47.4
200	35.83	38.1	37.39	38.1
300	33.5	33.91	32.64	33.35
450	26.28	26.8	27.29	26.79
600	16.6	16.48	15.87	16.32
800	7.52	7.3	7.24	7.35

**Table 13:** Analysis of Variance (ANOVA) table.

Source of Differences	Sum of Squares of Dispersion	Degree of Freedom	Average Squared	F	Distinctiveness
Temperature (between groups)	3213.26	5	642.65	1763.83	**
Error (within group)	4.37	12	0.36		

“**”, represents a very significant impact.

**Table 14:** Residual compressive strength of magnetite concrete under microwave heating.

Temperature (°C)	Residual Compressive Strength		Relative Residual Compressive Strength	
	Experimental (MPa)	Simulation (MPa)	Experimental (9%)	Simulation (%)
38	47.4	47.4	100	100
200	38.83	40.06	81.91	84.51
300	33.91	35.08	71.54	74.01
450	27.29	27.56	57.57	58.14
600	16.48	20.08	34.76	42.36
800	7.52	9.93	15.86	20.95

## Data Availability

The original contributions presented in this study are included in the article. Further inquiries can be directed to the corresponding author(s).
